# Role of microRNAs in Alcohol-Induced Multi-Organ Injury

**DOI:** 10.3390/biom5043309

**Published:** 2015-11-20

**Authors:** Sathish Kumar Natarajan, Joseph M. Pachunka, Justin L. Mott

**Affiliations:** Department of Biochemistry and Molecular Biology, University of Nebraska Medical Center, 985870 Nebraska Medical Center, Omaha, NE 68198, USA; E-Mails: joseph.pachunka@unmc.edu (J.M.P.); justin.mott@unmc.edu (J.L.M.)

**Keywords:** alcoholism, miRNA, lncRNA, teratogen, alcoholic liver disease, alcoholic pancreatitis

## Abstract

Alcohol consumption and its abuse is a major health problem resulting in significant healthcare cost in the United States. Chronic alcoholism results in damage to most of the vital organs in the human body. Among the alcohol-induced injuries, alcoholic liver disease is one of the most prevalent in the United States. Remarkably, ethanol alters expression of a wide variety of microRNAs that can regulate alcohol-induced complications or dysfunctions. In this review, we will discuss the role of microRNAs in alcoholic pancreatitis, alcohol-induced liver damage, intestinal epithelial barrier dysfunction, and brain damage including altered hippocampus structure and function, and neuronal loss, alcoholic cardiomyopathy, and muscle damage. Further, we have reviewed the role of altered microRNAs in the circulation, teratogenic effects of alcohol, and during maternal or paternal alcohol consumption.

## 1. Introduction to microRNA

MicroRNAs act to fine-tune the expression of a vast number of genes and regulate cell physiology and pathology. MicroRNAs are endogenous 19–23 nucleotide, small non-coding RNAs that modulate mRNA levels through decreased transcription or post-transcriptionally induced mRNA decay. The processing of microRNA was well described before [1,2]. Briefly, microRNA is mostly produced from RNA polymerase II transcribed genes in the nucleus; recently it has also been shown that RNA polymerase III can transcribe certain primary microRNAs (pri-microRNA). Pri-microRNA transcripts produced by RNA polymerase II are 5' capped and 3' poly‑adenylated in the nucleus. Pri-microRNA is then cleaved and processed by the RNase III enzyme, Drosha-DGCR8 (DiGeorge syndrome critical region 8), a microprocessor complex which results in ~70 nucleotide stem loop precursor microRNA (pre-microRNA). The pre-microRNA is exported from the nucleus to the cytoplasm by RanGTP-dependent dsRNA transport protein or exportin 5 [3]. Cytoplasmic RNase III enzyme, Dicer, in conjunction with TAR RNA-binding protein 2 binds to the pre‑microRNA and cleaves the terminal loop. The remaining duplex microRNA interacts with Argonaute protein and only one of the duplex strands (lead strand) binds to form microRNA-induced silencing complex. The other passenger strand is mostly degraded in the cytoplasm.

**Figure 1:**
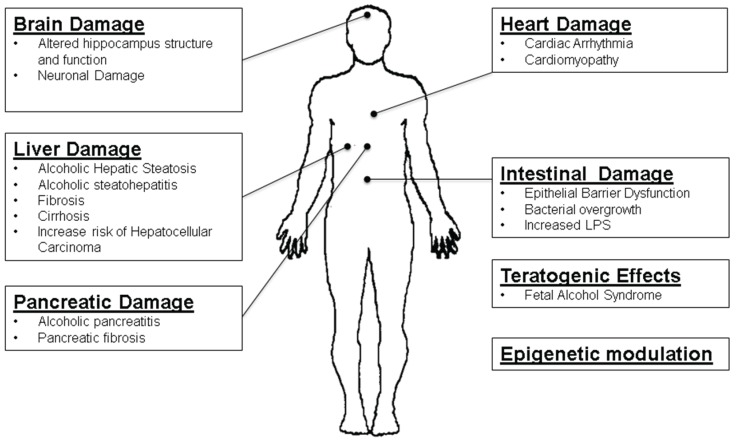
Ethanol Induces Damage to Vital Organs in the Human Body. Ethanol consumption induces liver cells to accumulate lipid droplets and predisposes to alcoholic steatohepatitis, cirrhosis and increases the risk for the development hepatocellular carcinoma. In the pancreas, chronic alcohol consumption results in pancreatitis and pancreatic fibrosis. In the intestine, ethanol induces epithelial barrier dysfunction and gram negative bacterial overgrowth resulting in an enhanced production of LPS for the pathogenesis of alcoholic liver injury. Ethanol also produces brain damage by altering hippocampus structure and function and neuronal loss. Chronic ethanol consumption also causes alcoholic cardiomyopathy and cardiac arrhythmias. Additionally, alcohol has been shown to act as a teratogenic agent and epigenetic modulator via altering non-coding RNA levels such as microRNA and lncRNAs.

MicroRNAs play a role in many different physiological and pathophysiological states including in alcoholic liver disease (ALD) [4] and microRNAs were suggested to be a master regulator of ethanol-induced multi-organ injury [5]. In this review, we will present mechanisms of alcohol-induced damage to vital organs like pancreas, liver, intestine, brain, and heart as shown in the Figure 1. Additionally, we will present evidence for a role of altered microRNAs in the vital organ dysfunction caused by acute or chronic alcohol consumption.

## 2. Alcohol Metabolism and Pancreatitis

Alcohol is mostly metabolized in the liver by the enzymes, alcohol dehydrogenase, acetaldehyde dehydrogenase and microsomal cytochrome p450-2E1 oxidase (CYP2E1). Alcohol metabolism leads to the generation of toxic metabolite, acetaldehyde, which reacts to various proteins to form acetaldehyde adducts resulting in improper protein functions [6]. Alcohol metabolism also results in an increased production of reactive oxygen species (ROS) through CYP2E1. Enhanced production of ROS initiates peroxidation of cellular lipids including fatty acids present in the cytoplasm and membranes [7]. Inhibition of CYP2E1 or CYP2E1 knockout in mice protects against ethanol-induced liver damage in rodent models [7]. However, inhibition of alcohol metabolism resulted in an increase in non-oxidative ethanol metabolites like fatty acid ethyl ester (FAEE) and phosphatidyl ethanol [8,9]. Increased levels of FAEE and phosphatidyl ethanol have been shown to directly correlate with alcoholic pancreatic injury and pancreatitis [8,9]. Recently, inhibition of FAEE synthase was shown to ameliorate ethanol-induced inflammation and acute pancreatic damage [9]. Ethanol exposed hepatoma cells, HepG2 were also shown to secrete increased amount of FAEE in to the medium in association with high density lipoproteins [10]. Chronic alcoholics have an increased level of FAEE in their hair and systemic circulation [11]. Increased FAEE accumulates in the pancreas for the initiation of acute pancreatic injury [8,9]. Increased FAEE has also been shown to affect intestinal epithelial barrier function by disrupting the tight junctions through ROS-dependent mechanisms [12].

### Altered microRNAs in Alcoholic Pancreatitis

Prolonged ethanol consumption induces alcoholic pancreatitis and pancreatic fibrosis [13,14,15]. Ethanol activates pancreatic stellate cells to produce pro-fibrogenic mediators like transforming growth factor-β (TGF-β) and platelet-derived growth factor, and extracellular matrix proteins for the initiation of pancreatic fibrosis and alcoholic pancreatitis [16,17]. Antioxidants like vitamin E were shown to prevent alcohol-induced pancreatitis in rodents [16]. Chronic ethanol feeding to mice induces an increase in the expression of miR-21, miR-199a-3p and miR-211 and down regulated miR-148a and miR-802 in the pancreas [18]. Similar changes in microRNAs were also noted in pancreatic stellate cells isolated from ethanol fed mice [18]. Further, increased miR-21 was suggested to regulate connective tissue growth factor as a positive feedback loop in chronic pancreatitis, where increased miR-21 increased connective tissue growth factors levels [18,19]. Ethanol was also shown to induce the expression of connective tissue growth factor in human hepatic stellate cells [20]. Together, microRNAs are involved in the activation of pancreatic stellate cells during alcoholic pancreatitis and pancreatic fibrosis. Further studies are needed to elucidate whether a miR-21 inhibitor can prevent alcoholic pancreatitis and pancreatic fibrosis.

## 3. Circulating microRNAs in ALD

Circulating microRNAs have been studied as biomarkers for ALD. Emerging evidence has shown that microRNAs are stable in circulation and they are also resistant to circulating RNases that are abundant in blood [21]. It was earlier thought that circulating microRNAs are only released along with microvesicles or exosomes providing RNase protection. But recent studies have shown that many ribonucleoprotein complexes like nucelophosmin-1, argonaute1 and 2-bound microRNAs were released in to the blood and these RNA-binding proteins protect extracellular degradation of microRNAs [22,23,24,25,26]. Rats fed an ethanol diet showed increased circulating levels of miR-122 and miR-155 in exosomes [27]. Ethanol-induced increase in circulating miR-122 was abolished in toll-like receptor 4 (TLR4) and NADPH oxidase knockout mice suggesting a critical role for TLR4 and NADPH oxidase in the release of miR-122 [27].

Circulating miR-122 was suggested as a biomarker for liver injury similar to serum ALT and not as a marker for disease progression. However, circulating microRNAs that are upregulated in ALD were also found to be increased in other liver diseases like drug-induced liver toxicity and non-alcoholic fatty liver disease [28]. An animal model of alcoholic steatohepatitis showed 25 different microRNAs were upregulated in the circulation and seven microRNAs were down regulated. Among these only four of them (miR-185, miR199a-3p, miR-214 and miR-490) were shown to be similarly altered in liver tissue and circulation [29]. This suggests that circulating microRNAs are not solely from liver damage; another major source of circulating microRNAs was blood cells [30,31]. A recent analysis of patients with diabetes showed that there was a difference in the levels of circulating microRNAs between platelet-rich plasma and platelet-poor plasma suggesting that platelets are substantial contributors of microRNA in the circulation [32,33]. An epidemiological study has shown that alcohol consumption was inversely associated with platelet activation and aggregation [34]. Further studies are needed to elucidate the role of platelet microRNAs in ALD.

### 3.1. Ethanol Alters Monocyte Function via Circulating microRNAs

Patients with ALD show elevated levels of plasma IL-17 and CCL2. IL-17 is an inflammation-promoting cytokine and CCL2 is a chemotactic protein that help attracts monocytes to the site of inflammation [35,36,37]. Chronic alcoholics also had elevated levels of IL-17-producing T-helper lymphocytes. Hepatic stellate cells have the receptor for IL-17 and increased IL-17-producing T-helper lymphocytes were suggested to be recruited to the liver from the circulation in patients with chronic alcoholism [37,38,39]. Binge alcohol consumption was recently shown to increase exosomal miR-122 and miR-155 in the circulation [39]. Further, primary hepatocytes also showed an increase in the release of miR-122-containing exosomes [39]. Exosomes-isolated from hepatocytes were added to the human monocyte-derived cell line, THP-1, along with LPS to mimic ALD patient’s circulation [39]. Hepatocyte exosomes plus LPS treatment to monocytes increased cytokine and chemokine levels such as TNF-α, IL1-β, and CCL2 [39]. These increased pro-inflammatory cytokines and chemokine levels due to the treatment of hepatocyte-exosomes were abolished with anti-sense miR-122 inhibitor treatment in the THP1 cells [39]. Clearly, circulating miR-122 derived from injured hepatocytes alters monocyte and T-helper lymphocyte function to promote inflammation in ALD.

### 3.2. Ethanol Abuse and Bone Marrow Depression

Alcoholism is a significant contributing factor for vitamins B1, B3, B6, B7, C, D and folate deficiency [40]. Chronic ethanol consumption has been shown to exert its toxic effect on bone marrow and results in the impairment of blood cell production or bone marrow depression. The vitamin deficiency further exacerbates the bone marrow depression [41]. Chronic alcohol consumption was shown to damage red blood cell precursors and resulted in producing enlarged RBCs. Several studies have also reported macrocytosis (enlarged RBCs) and osteoporosis in chronic alcohol abuse patients [41,42,43]. Further, ethanol and acetaldehyde can directly inhibit the osteoblastogenic potential of the bone marrow and decreases bone formation [44]. Similarly, alcohol also increases bone mass degradation, density and negatively regulates bone microarchitecture [45]. Autologous bone marrow-derived stem cell transplantation was shown to improve liver function in patients with ALD and decompensated alcoholic cirrhosis in randomized clinical trials [46,47,48]. Together, chronic alcoholics develop an alcoholic bone disease and the potential therapeutics for this toxic effect on bone marrow has been recently reviewed [42,49]. The role of microRNAs in bone-related disease has been reported. For example, miR-29a, miR-31, miR-133a, and miR-378 can modulate osteogenic differentiation of mesenchymal stem cells by targeting osteoclast-specific genes [50]. Additionally, circulating microRNAs like miR-328-3p, let-7g-5p were shown to be decreased with osteoporotic bone fracture [51]. However, the role of microRNAs in bone marrow depression during alcoholism or ALD has not been studied and needs further investigation.

### 3.3. Pitfalls of Circulating microRNA Analysis

Circulating microRNAs have been proposed to serve as a biomarker and prognostic marker for alcoholism, ALD and alcoholic steatohepatitis [29,52]. Also, circulating microRNAs were suggested to be a blood marker for liver inflammation, fibrosis and cancer detection [53,54]. However, there are potential pitfalls of measuring microRNAs in the circulation. As discussed above the role of blood cell microRNA changes during alcoholism need to be considered. Another pitfall is that candidates for use in microRNA normalization, for example, small nuclear and small nucleolar RNAs like RNU4, RNU6b (U6) and RNU48 were highly variable in healthy populations [55]. Notably, U6 is downregulated in the serum of patients with liver fibrosis [56]. In contrast, U6 levels in the circulation were upregulated in patients with critical illness and sepsis [56]. The use of miR-16 as an internal control for circulating microRNAs has also been reported [57]. However, miR-16 and miR-15b levels were shown to be susceptible to hemolysis of blood samples [56,57]. Avoiding several technical variability factors like isolation procedures, storage, multiple freeze-thaw cycles of a sample and the use of an appropriate amount of sample and enzymatic reactions were suggested to minimize variations and difference [55]. For example, a report showed that low GC content microRNAs were lost in the extraction protocol, when a small number of cells were used compared to a high number of cells [58]. Additionally, spiking of non-human microRNAs like *Caenorhabditis elegans* cel-miR-67 as an external control during RNA extraction, cDNA synthesis, and PCR amplification were suggested to be helpful to track the loss of microRNAs during extraction and processing [59]. For internal control of circulating microRNA analysis, various strategies have been proposed in a case-by-case study [55]. From the analysis of healthy volunteers, it has been proposed that miR-24, miR-126, and miR-484 were stable microRNAs [55].

## 4. Liver microRNAs in Alcoholic Liver Disease

Alcohol-induced liver damage includes a spectrum of complications like steatosis, alcoholic steatohepatitis, fibrosis, cirrhosis, and increased risk for hepatocellular carcinoma. MicroRNAs in the liver are regulated by exposure to an ethanol-containing diet [52,60]. Mice fed a Lieber-DeCarli alcohol diet were studied for hepatic microRNA content as an initial screen for microRNAs that may be involved in ALD. Most microRNAs (~98%) were not altered, while approximately 1% were increased by alcohol feeding and 1% were decreased. The increased microRNAs include miR-320, miR-486, miR-705, and miR-1224. Decreased microRNAs were miR-27b, miR-182, miR-183, miR-199a-3p, miR-200a, miR-214, and miR-322 [61]. A separate study performed by gavage of alcohol by a gastrostomy tube found that ethanol increased miR-21, miR-34a, miR-137, miR-409-5p, miR-509-3p, and miR-882 and decreased levels of let-7 family members (let-7a, -7b, and -7g), miR-122, miR-127, miR-181a, miR-181b, miR-192, and miR-871 [62]. The lack of overlap in these microRNA sets is notable and may reflect the difference in a liquid ethanol-containing diet *versus* ethanol gavage. In addition, alcohol decreased the expression of miR-199 in human and rat liver sinusoidal endothelial cells [63]. Targets of miR-199 were endothelin-1 and HIF1α, loss of miR-199 expression increased endothelial HIF1α levels [63]. Increased HIF1α functions as a survival signal and induced lipid accumulation in alcoholic fatty liver disease [64] and has been reviewed before [65].

Patients with ALD, and animal models of ALD, have increased levels of hepatic and circulating tumor necrosis factor-α (TNF-α) [66,67,68]. In normal macrophages, it has been suggested that the 3'-untranslated region of TNF-α mRNA inhibits its own translation [69]. Alcohol also induced the expression of miR-155 and TNF-α in Kupffer cells and macrophages [63,70]. Upregulation of miR-155 in macrophages is via the activation of NFκB [70]. Inhibition of miR-155 using anti-sense-miR-155 prevented the production of TNF-α in macrophages and further, miR-155 increased the half-life of TNF-α mRNA [70]. Alternatively, ethanol induced the translational initiation and stabilization of TNF-α mRNA by increasing the expression of AU-rich binding protein, HuR. Further, miR-132 and miR-155 were elevated in the isolated hepatocytes and Kupffer cells from alcohol fed animals [70]. MiR-132 targets NAD-dependent deacetylase, SIRT1 and p65 subunit of NFkB which can result in decreased NFkB activity [71]. Interestingly, a recent study showed that hepatocytes isolated from miR-155 knockout mice were resistant to Fas-induced apoptosis [72]. Myeloid cell leukemia-1 (MCL1) was found to be upregulated in the ethanol-fed liver studied from miR-155 knockout mice, suggesting that MCL1 is a direct target of miR-155 [72]. Elevated levels of miR-155 after chronic ethanol could also function to induce apoptosis of hepatocytes and other liver cells in ALD [72,73]. Together, liver microRNAs are altered in ALD.

Chronic alcohol feeding of mice showed upregulation of miR-33, miR-34a, and miR-217 in the liver and these microRNAs were also elevated in ethanol-exposed mouse AML-12 hepatocytes. Alcohol dehydrogenase, but not aldehyde dehydrogenase was found to be critical for the increased expression of miR-217. Further, SIRT1 was identified as a target gene for miR-217 in the liver. Ethanol treatment decreased the expression of SIRT1 and a similar decrease in SIRT1 was observed in hepatocytes transfected with miR-217 mimic [74]. Ethanol treatment of miR-217-transfected hepatocytes exacerbated the decrease in the levels of SIRT1 protein [74,75]. Downstream mediators of SIRT1 were SREBP-1 (sterol regulatory element-binding protein 1) and peroxisome proliferator-activated receptor1-alpha (PGC-1α) [75]. Loss of SIRT1 increased the level of acetylated SREBP-1 and acetylated PGC-1α for enhanced lipogenesis in the alcoholic liver and has been reviewed before [75]. Additionally, lipin-1, a phosphatidic acid phosphohydrolase, which converts phosphatic acid into diacyl glycerol was also found to be down-regulated by miR-217 [76]. In contrast, ethanol can directly induce the expression of lipin-1 in the liver via inhibition of AMP kinase and activation of SREBP-1 on the lipin-1 promoter [77]. In ethanol fed mice, miR-33 and miR-34a were also increased, though not in cultured cells [74]. MiR-33 was found to decrease VLDL secretion by repressing N-ethylmaleimide sensitive factor, a protein involved in SNARE-dependent intracellular lipoprotein trafficking [78,79]. Importantly, ethanol feeding increased miR-33 expression and decreased VLDL secretion [4,79]. Defective VLDL secretion has been implicated in the pathogenesis of alcoholic hepatic steatosis [80].

### 4.1. Liver miR-34a in ALD

MiR-34a is well known for its dual function as pro- and anti-apoptotic microRNA. Expression of miR-34a was found to be increased in hepatocytes and cholangiocytes with LPS plus ethanol exposure and in the livers of alcohol fed animals. Ethanol induced hypo methylation in the promoter regions of miR-34a thereby increasing its expression. Increased miR-34a was shown to target and represses caspase-2 and SIRT1 proteins [62]. This represents a second SIRT1-targeting microRNA that is elevated with ethanol feeding in rodents (miR-34a and miR-217). Here, increased miR-34a in the liver was suggested to promote survival of hepatocytes, hepatic stellate cells and cholangiocytes [62]. Ethanol was also shown to export SIRT1 from the nucleus to the cytoplasm. Ethanol-induced SIRT1 nucleo-cytoplasmic shunting was suggested to be due to the generation of ROS and acetaldehyde, an ethanol metabolic byproduct [81]. SIRT1 is a redox sensitive enzyme and gets post-translational modifications with the exposure of oxidants and acetaldehydes resulting in decreased enzyme activity and undergoes proteasome degradation [82]. Further studies are needed to determine the exact role of SIRT1 and miR-34a in the progression of ALD.

### 4.2. Role of miR-21 in ALD

Liver regeneration and hepatocyte proliferation after 2/3 partial hepatectomy was shown to be dependent on miR-21. MiR-21 targets *Btg2*, a cell cycle inhibitor. Normally, *Btg2* prevents the activation of forkhead box protein M1 (FoxM1), a protein important for hepatocyte DNA synthesis after partial hepatectomy [83]. Studies have shown that prolonged ethanol feeding enhanced miR-21 expression in the liver [84,85]. Ethanol fed animals showed a dramatic increase in miR-21 12h after partial hepatectomy compared to control-fed animals [84]. Further, 5 weeks of ethanol (5% *v*/*v*) to mice was also shown to induce the levels miR-21 and miR-34a [85] and a similar increase miR-21 was also observed in the liver of patients with ALD [85].

Inhibition of miR-21 was well known to induce apoptosis in most cancer cells including ovarian cancer cells [86,87]. Overexpression of miR-21 in normal hepatocytes results in resistance to ethanol-induced apoptosis suggesting an important role for this microRNA [85]. The mechanism of miR-21 induction with ethanol exposure in the liver is via activation of IL-6/STAT3 signaling pathway [73,85]. Ethanol-induced activated STAT3 binds to the miR-21 promoter region for transcriptional up-regulation of miR-21 primary transcripts [73,85]. Additionally, miR-21 was shown to target the 3' UTRs of FAS ligand G (FASLG) and death receptor 5 (DR5) [85]. Treatment of ethanol to primary hepatocytes, hepatic stellate cells and HepG2 cells was also shown to decrease the expression of FASLG and DR5 proteins via increasing miR-21 levels [85]. Together, miR-21 is induced with ethanol exposure via IL-6/STAT3 signaling and targets extrinsic apoptotic mediators like FASLG and DR5 for the prevention of liver cell apoptosis and could favor liver regeneration in ALD.

## 5. Gut-Liver Axis in the Pathogenesis of ALD

Alcohol consumption is well known to damage intestinal barrier function which results in the translocation of gut-derived lipopolysaccharide (LPS) into the portal and systemic circulation. LPS is a cell wall structural component of gram negative bacteria [39]. Source for LPS from the gut is due to the loss of tight junction proteins in the intestine epithelial cells. LPS can simply diffuse into circulation or can be transported through mesenteric lymph system from the intestine and enter into systemic circulation [88,89]. Hepatocytes and Kupffer cells in the liver remove LPS from the circulation or lymphatic system. Detoxification of LPS in the liver is compromised in ALD. Alcohol-induced increased LPS from the gut due to bacterial overgrowth results in LPS accumulation to other organs like spleen and brain in ALD [90,91]. Several studies have demonstrated that patients with chronic alcoholism have elevated levels of LPS in the systemic circulation [88,89,90,91]. In animal models of ethanol administration there is a 5–15 fold increase in LPS in the portal and systemic circulation [92].

Ethanol feeding was shown to shift the gut microbiome towards an increase in gram negative bacteria-dependent endotoxemia and increased intestinal permeability [93]. Metagenomic deep sequencing analysis of gut microbes in alcoholics further reveals that there was a significant increase in *Alcaligenes* genus, gram negative bacteria [93]. Intestinal *Alcaligenes* growth is ideal only in alkaline pH and it was found that alcohol feeding increases cecal pH, favoring *Alcaligenes* overgrowth [93]. Supplementation of non-absorbable antibiotics, dietary fiber, probiotics and saturated long-chain fatty acids (LCFA) was shown to prevent ethanol-induced intestinal bacterial translocation and decreased liver damage due to ethanol intoxication [93,94]. Supplementation of *Lactobacillus* GG also prevents loss of tight junction proteins in the intestine, endotoxemia and hepatic injury caused due to ethanol feeding to mice [94].

Intestinal bacteria ferment indigestible dietary fibers into short chain fatty acids (SCFA) and gut microbes also produce many nutrients (polyunsaturated fatty acids, gamma-amino butyric acid, histamine) and essential vitamins like vitamin B and vitamin K [95]. Chronic alcoholics show an increase in oral-to-cecal transit time which might help bacterial overgrowth and increases bacterial metabolites [95]. A recent metabolomics study in mice fed an ethanol diet for 8 weeks showed increased levels of acetic acid, an end product of ethanol metabolism [95]. In contrast, SCFAs like propionic acid, isobutyric acid, and butyric acid were decreased in ethanol-fed animals suggesting a loss of SCFA producing bacteria [95]. Butyrate, a SCFA is a well-known fuel for colonic epithelial cells [96]. Butyrate has also been shown to protect intestinal barrier by increasing the assembly of tight junction through several mechanisms including AMP kinase activation [96,97]. Ethanol-induced loss of butyrate production could alter the repair response for the intestinal barrier dysfunction. Further, decreased SCFAs with ethanol could also decrease immune-protective effect against bacterial infection in the gut [98]. In summary, ethanol-induced bacterial overgrowth, dysbiosis towards an increase in gram negative bacteria, endotoxemia, and intestinal permeability contributes to the pathogenesis of alcoholic liver injury.

### 5.1. Altered Intestinal microRNAs in ALD

The role of microRNA in the pathogenesis of intestinal injury with ethanol was reported. Here we will briefly discuss the functions of microRNAs that are altered with ethanol consumption. Chronic ethanol administration induces the expression of miR-155 in the intestine [73]. Similar to the effect observed in the liver, ethanol-induced upregulation of miR-155 was shown to stabilize and increase the half-life of TNF-α messenger RNA and its molecular target Reg3b [73]. Reg3b is a C-type lectin secreted into the intestinal lumen for its antimicrobial activity against gram negative bacteria to prevent bacterial translocation across the lumen [99]. Acute ethanol exposure was shown to increase the levels of Reg3b protein in the small bowel [73]. Post-translational modifications of Reg3b were suggested as a mechanism for increased Reg3b protein levels with acute ethanol exposure [85]. Recently, Reg3b Knockout mice were found to be more prone to gram negative bacterial infection [99]. Mice deficient in miR-155 did not show an increase in Reg3b protein levels with chronic ethanol exposure suggesting the role of Reg3b and miR-155 in alcohol-induced intestinal dysfunction [73].

MicroRNA-155 has been shown to be involved in inflammation, immunity and also in hematopoiesis [100]. MiR-155 was shown be increased in many diseases that involve inflammation including ulcerative colitis [73]. Increased miR-155 in patients with colitis was shown to decrease suppressor of cytokine signaling 1 (SOCS1) expression via targeting its 3'UTR [101]. SOCS1 was down regulated in the liver and Kupffer cells isolated from ethanol-fed mice suggesting that ethanol-induced miR-155 may negatively regulate SOCS1 in the liver [102]. Involvement of SOCS1 in the intestine with ethanol has not been determined yet, but, miR-155 targeted and downregulated other inflammatory markers like Src homology 2-containing inositol phosphatase 1 [73,103]. These observations indicate miR-155 may be involved in small bowel inflammation with chronic ethanol exposure [73,103].

### 5.2. microRNAs and Intestinal Tight Junctions in ALD

Expression of miR-212 was found to be highly abundant in intestine and colon compared to other organ like heart, liver, and kidney [104]. Increased levels of miR-212 in the intestine were reported with alcohol feeding and this increased miR-212 targets tight junction proteins like Zona Occludens-1 (ZO-1). Colonic tissues from patients with ALD showed enhanced expression of miR-212 and also showed a concomitant decreased ZO-1 protein level. This study suggests that increased miR-212 in the small intestine and colon targets tight junction protein and negatively affects intestinal barrier function in ALD [104]. Further studies are needed to clarify the mechanism of miR-212 upregulation in the intestine and colon of ALD.

MiR-122 is a liver specific microRNA, but has been found at low levels in non-hepatocytes. TNF-α was shown to induce the expression of miR-122 in colon-derived Caco-2 cells and enterocytes [105]. TNF-α-induced miR-122 in enterocytes targets 3' UTR of occludin transcript, a tight junction protein [105]. Ethanol-fed mice also showed an increase in the expression of miR-122 in the intestine and decreased expression of occludin and claudin-1 in the intestine thereby inducing barrier dysfunction [106]. Probiotic supplementation of *Lactobacillus rhamnosus* GG was also shown to protect intestinal barrier dysfunction by decreasing the expression of miR-122 in chronic ethanol-fed mice [106]. However, bacterial translocation is not unique to ALD; we have shown that liver cirrhosis induces alterations in the luminal glycosylation pattern and bacterial overgrowth leading to bacterial translocation [107,108,109]. Whether intestinal bacterial translocation in cirrhotic animals is mediated by miR-122 is unknown. In summary, altered microRNAs in the intestine due to ethanol exposure play a role in the pathogenesis of ALD by increasing the expression of pro-inflammatory cytokines, and decreasing tight junction proteins, favoring intestinal permeability and endotoxemia.

## 6. Brain Injury and microRNAs with Alcohol Consumption

Brain injury due to alcohol consumption has been well documented. Here we will review the important role of microRNAs in alcohol-induced injury to neurons. Ethanol exposure to neurons in culture decreased the expression of miR-9, miR-21, miR-153 and miR-335 [110,111,112]. A recent study also revealed that miR-9, miR-29a, miR-29b and miR-133 were decreased in cerebellum granule neurons isolated from mice exposed to ethanol. Ethanol decreased the expression of miR-29b in mouse cerebrum. Experimental validation confirmed that overexpression of miR-29b protected against ethanol-induced neuronal cell apoptosis [113]. Also, ethanol induced the expression of SP1 transcription factor, murine homolog of human PACT (RAX or dsRNA-binding protein and an activator of PKR) and phosphorylation of dsRNA-activated protein kinase (PKR, a serine threonine protein kinase) involved in inflammation and neuronal apoptosis [113,114]. Ethanol-induced enhanced expression of SP1 and RAX, and increased phospho-PKR were completely blocked in miR-29b overexpressing neurons [113]. In addition, ethanol also increased the expression miR-34a that acted as pro-apoptotic microRNA [113]. Alcohol increased the expression of miR-9 when analyzed using single cell based PCR method [115]. Increased miR-9 in the supraoptic neurons and striatal neurons targeted calcium- and voltage-activated potassium channel in the brain [115,116].

MiR-132 and miR-212 are encoded by a single gene [103] and the role of miR-212 in the intestine barrier dysfunction is discussed in “microRNAs and intestinal tight junctions in ALD” section. MiR-132 and miR-155 levels were elevated in the brain and livers of mice administered an ethanol-containing diet [103]. The mechanism for increased expression of brain miR-132 and -155 was through activation of TLR-4 [103]. Pro-inflammatory cytokines like TNF-α, MCP1, and IL-1β were upregulated in the cerebellum of chronic ethanol fed mice [103]. Ethanol-induced increase in pro-inflammatory cytokines expression in the brain was due to an increase in the expression of miR-155. Ethanol did not alter brain pro-inflammatory cytokine levels in mice deficient for miR-155 [103]. In summary, levels of microRNAs were altered in the brain with ethanol consumption contributing to the brain injury observed with alcoholics.

### 6.1. Binge Drinking Alters Hippocampal microRNA

Emerging evidence indicates that a majority of adolescent drinkers consume alcohol in binge episodes [117,118]. A recent survey among high school students showed shocking information that 21% of high school students were binge drinkers in the United States [117]. Adolescent alcohol binge drinking was suggested to be associated with long-term memory loss, depression, behavioral problems, and poor stress response [117,118,119]. Rodents exposed to binge ethanol during peripubertal stage exhibit damage to hippocampal structure and function and reduced adult neurogenesis resulting in impaired memory and neuropsychiatric illness [120].

MicroRNAs were suggested to play a role in hippocampal structure and function. Repeated binge ethanol exposure in adolescent rats was shown to dramatically increase the expression of miR-10a-5p in the hippocampus [119,121,122]. In addition, proteins involved in microRNA processing like Drosha and Dicer were also found to be decreased in the ventral and dorsal hippocampus in ethanol exposed adolescent rats [119]. Rats exposed to acute-binge ethanol during puberty showed decreased expression of miR-26a and miR-495 in the dorsal and ventral hippocampus [119]. The ethanol-induced decrease in miR-26a and miR-495 was suggested to increase target mRNAs, brain-derived neurotropic factor (BDNF) and SIRT1, a protein deacetylase [119]. However, increased expression of BDNF and SIRT1 in the hippocampus was recently demonstrated to offer protection to hippocampus and neurons [123,124,125]. Further studies are needed to confirm the exact role of miR-10a-5p, miR-26a and mi-495 in hippocampal damage during ethanol exposure.

### 6.2. Neuronal Damage and microRNA

The effects of alcoholism on brain white matter shrinkage and neuronal loss in frontal cortex have been known for more than three decades [126,127]. Within last few years, a role for microRNAs in alcohol-induced brain damage has been proposed and identified. A comprehensive analysis of microRNA levels in the frontal cortex of patients with alcoholism showed that 35 microRNAs were upregulated including let 7 family members, miR-34c, miR-146a, miR-194, miR-203, and miR-369. This study further analyzed mRNA expression [128]. Several of the microRNAs that targeted mRNAs involved cell cycle, differentiation, signaling and nervous system development were found to be down regulated in human alcoholics [128]. In a human neuroblastoma cell line, *in vitro* long term ethanol exposure (72 h) results in a dramatic increase in miR-302b and miR-497. Both miR-302b and -497 can target BCL2; increased levels of miR-497 were shown to be critical for mitochondria-dependent neuronal apoptosis [129]. Increased miR-302b was suggested to target cyclin D2 and induced mitochondria-independent apoptosis of neurons during alcohol exposure [129]. In addition, the pro-apoptotic microRNA, miR-34a was also shown to be upregulated in neurons exposed to ethanol [113,129]. Similarities and differences in these two pioneer studies on ethanol-induced brain microRNAs were discussed and reviewed in detail before [130].

### 6.3. Long Noncoding RNA and Ethanol

Long noncoding RNAs (lncRNAs) range between 200 nucleotides to 100 kb and mostly do not have a potential to encode protein [131]. A recent update from LNCipedia, a database for annotated lncRNA sequence, shows that there were more than 111,000 human annotated lncRNAs [132]. lncRNAs are highly conserved and involved in diverse physiological processes including neuronal development [133]. The study of lncRNAs during ethanol toxicity to the brain is in its infant stage. However, there was a large increase in the expression of lncRNA, *MALAT1* (metastasis associated lung adenocarcinoma transcript 1) in the cerebellum, hippocampus and brain stem of human alcoholic brain sections obtained from postmortem brain [134]. Increased expression of *MALAT1* was also observed in rat brain tissues after acute exposure to ethanol [134]. However, the exact mechanism of increased *MALAT1* expression in human alcoholics was not clear. Oxytocin is known to induce the expression of *MALAT1* in a neuroblastoma cell line [135], and plasma oxytocin was elevated in human alcoholics [136]. Oxytocin levels stayed elevated ever after a month of abstinence [136]. This raises the question; does oxytocin play a critical role in the induction of *MALAT1* expression? Recently, a separate study in patients with alcoholism showed 2–5 fold increased expression of 11 lncRNAs (NCRNA00051, NCRNA00256A, NCRNA00256B, NCRNA00250A, NCRNA00245, NCRNA00176, NCRNA00116, NCRNA00173, NCRNA00175, NCRNA00230B, and NCRNA00107) [137]. Four other lncRNAs and were shown to be downregulated in postmortem prefrontal cortex of human brain [137]. The functions of many lncRNAs are still unknown, including the 15 lncRNAs identified in the patients of above described study [137]. Further studies are needed to elucidate the exact function and role of these lncRNAs in brain damage observed in human alcoholics.

### 6.4. Teratogenic Effect of Ethanol

Ethanol consumption during pregnancy can be associated with fetal alcohol syndrome. Fetal alcohol syndrome is a spectrum of symptoms ranging from abnormal cardiac development, neuronal loss, abnormal facial features, growth defect and damage to central nervous system [138]. Ethanol was shown to have teratogenic effect on the fetal mouse brain and several microRNAs were reported to be involved in this teratogenic effect [139]. Prenatal ethanol exposure was shown to cause mental retardation in offspring with impaired locomotor function. Prenatal ethanol exposure was also shown to up-regulate several microRNAs (miR-9, miR-10a, miR-10b, miR-30a-3p, miR-145, and miR-152) in the fetal brain [139]. Among them, miR-10a and 10b were shown to target homeobox transcription factor expression [140,141]. miR-10a and miR-10b also target TBX5, a T-box transcription factor involved in embryonic cardiac development [142]. Supplementation of folic acid prevented the increase in miR-10a caused due to ethanol in the fetal brain [139]. Folic acid treatment also protects cardiac birth defects in mice due to fetal alcohol syndrome [143]. Ethanol ingestion was found to dramatically increase retinol and retinoic acid receptor levels in the fetal embryo [144]. Retinoic acid receptor antagonist has been shown to inhibit the expression of miR-10a in pancreatic cancer cells [140]. Further, ethanol intoxication increases the levels of *all-trans* retinoic acid levels in the embryo [122,144]. Ethanol-induced increase in the levels of *all-trans* retinoic acid in the brain and hippocampus were due to an increase in the expression of retinoic acid synthesizing enzymes [122]. It is tempting to speculate that increased retinoic acid could be responsible for the observed increase in the expression of miR-10a and miR-10b during fetal alcohol syndrome. Further studies are needed to confirm this hypothesis of ethanol-induced retinoic acid activating retinoic acid receptor (RAR) to enhance miR-10 expression in fetal alcohol syndrome.

### 6.5. Maternal Ethanol Consumption

Maternal ethanol consumption and microRNA changes during fetal alcohol syndrome has been extensively studied and reviewed before [5,110,111,112,121]. Recently, ethanol was shown to decrease the expression of miR-335 and miR-9 in the fetal neural stem-cell population and this altered microRNA expression was suggested to be due to hypo-methylation was extensive studied and reviewed before [112,121,145,146,147,148]. Prenatal exposure of alcohol results in life-long problems, e.g., a recent study in mice exposed to ethanol before birth showed increased miR-26b in the brain of the next generation of mice. Increased miR-26b targets and decreases the levels of cannabinoid receptor 1 (CB_1_R) [149]. CB_1_R is a G-protein coupled receptor and was shown to be abundantly present in the mammalian brain. CB_1_R confers neuroprotection against excitotoxicity by inducing brain-derived neurotropic factor (BDNF) via PI3K-protein kinase B(Akt)- mammalian target of Rapamycin complex 1 axis [150]. Human alcoholics also have shown a decrease in CB_1_R in their brain as evidenced by positron emission tomography analysis. This decreased brain CB_1_R was not reversible even a month after self-withdraw of alcohol consumption [151]. Further studies are needed to confirm whether miR-26 plays a role in decreasing CB_1_R levels in the brain of patients with alcoholism or acute alcohol exposure.

### 6.6. Paternal Ethanol Consumption and Epigenetics

Paternal ethanol exposure was also shown to alter the mental and physical health of an offspring [152]. Several studies have confirmed that paternal ethanol consumption results in hyperactivity and impaired cognitive skills in children [152,153]. Paternal alcohol consumption and epigenetic mechanisms which affects the F1 generation have been recently reviewed [153,154,155]. Paternal ethanol consumption was shown to decrease cytosine methyltransferase transcript levels in sperm [156]. Also, mammalian sperm-derived miR-34c has been shown to be critical for the zygote’s first cell division by targeting BCL-2 protein expression [157]. Here BCL-2 was suggested to have an anti-proliferative function by negatively regulating cyclin-dependent kinase inhibitor, p27 [157,158]. Paternal alcohol consumption and microRNA changes in the sperm have not been studied yet. However, exposure of male mice to stress or paternal stress increases several sperm microRNAs such as, miR-29c, miR-30a, miR-30c, miR-32, miR-193, miR-204, miR-375, miR-532-3p and miR-698 [159]. Similarly, high fat diet-induced obese male mice also showed global hypomethylation of sperm DNA and exhibited altered microRNAs levels [160,161]. Alcohol-induced changes in paternal microRNAs should be investigated.

## 7. Alcoholic Cardiomyopathy

Alcohol consumption also affects other vital organ like heart. Epidemiological evidence suggests that heavy drinking predisposes to the development of cardiac arrhythmia [162], cardiomyopathy and/or even sudden cardiac death [163,164,165,166]. Chronic alcohol intake results in increased cardiac remodeling by reducing gap junction proteins like connexin 43, decreased size of cardiomyocytes and altered myocardial conduction. Chronic alcohol-fed mice displayed mitochondrial damage in cardiomyocytes [167]. Further, acute ethanol exposure was also shown to decrease atrial current densities [168]. MicroRNAs play an important role in cardiac function and ageing, e.g., miR-34a was shown to be upregulated in the aged heart, contributing to cardiomyocyte cell death which results in an age-related decrease in cardiac function [169]. Additionally, ethanol feeding increased the levels of beclin 1, an autophagic marker and triggered the formation of autophagosomes in cardiomyocytes and results in myocardial contractile dysfunction through autophagy [170]. Similarly, ethanol exposure decreased the expression of miR-30a, a target of beclin 1 in cardiomyocytes [170]. A recent study profiled the microRNA signature in plasma from patients with alcoholic cardiomyopathy compared to a healthy population [171]. Patients with alcoholic cardiomyopathy showed changes in several microRNAs (miR-138, miR-485-5p, miR-506, miR-512-5p, miR-548-3p, and miR-4262) and suggested to be involved in the development of cardiac dysfunction [171]. Among these microRNAs, miR-138 was recommended as a biomarker for early diagnosis of alcoholic cardiomyopathy. Further studies are needed to elucidate the functional role of these microRNAs and to confirm that they are indeed from cardiac tissue.

### Role of microRNAs in Alcohol-Induced Muscle Injury

Chronic alcoholism also contributes to muscle myopathy and muscle weakness termed as alcoholic myopathy. Interestingly, females are more susceptible to alcoholic myopathy and cardiomyopathy than male counterparts [172,173]. Alcoholic myopathy was shown to be highly associated with the malnutrition not with the severity of liver disease, duration of alcohol abstinence or neuropathy. Ethanol inhibits muscle function by blocking muscle membrane channels, sodium, potassium pumps and impairs muscle mitochondrial function [174,175]. A recent study in rats fed with *ad lib* liquid 6.7% ethanol-containing diet for 3 weeks showed increased miR-127, miR-136, miR-378 and miR-150.3p. Mir-92b, miR-96-5p, and miR-192-5p were downregulated in muscle with ethanol feeding. However the main objective of this study was to compare brain and serum microRNA [176]. Further studies are needed to determine the exact functions of these altered microRNAs in muscle injury with ethanol consumption.

## 8. Conclusions

**Table 1:** Ethanol exposure induces the levels of microRNAs in various organs. Altered microRNAs were listed by affected organs, and their known target proteins were included in this table.

Affected Organ	Upregulated microRNA	Targets	References
**Pancreas**	miR-21	Connective tissue growth factor	[18,19]
	miR-199a-3p		[18]
	miR-211		[18]
**Liver**	miR-21	Btg2, IL-6, STAT3	[24,36,45,46,47]
	miR-33	VLDL	[37,47]
	miR-34a	Caspase-2, SIRT1	[24]
	miR-132	p65 subunit of NFkB	[34]
	miR-137		[24]
	miR-155	TNF-α, MCL1,SIRT1	[25,32,35]
	miR-185		[84]
	miR-199a-3p		[84]
	miR-214		[84]
	miR-217	SIRT1, Lipin-1	[39]
	miR-320		[23]
	miR-409-5p		[24]
	miR-486		[23]
	miR-490		[84]
	miR-509-3p		[24]
	miR-705		[23]
	miR-882		[24]
	miR-1224		[23]
**Intestine**	miR-122	TNF-α	[71,72]
	miR-155	FASLG, DR5, TNF-α, Regb, Src homology domain 2-containing inosital phosphatase	[36,67,68,69]
	miR-212	ZO-1	[70]
**Circulation**	miR-122	TLR4, NADPH Oxidase	[82]
	miR-155		[36,50,78,82]
	miR-185		[84]
	miR-199a-3p		[84]
	miR-214		[84]
	miR-490		[84]
**Brain**	Let 7 Family members		[109]
	miR-9		[120]
	miR-10a, miR-10b	TBX5, Homeobox	[97,99,120,121,122,123]
	miR-21		
	miR-26b	CB1R	[130]
	miR-29b	SP1, RAX, Phospho-PKR	[91]
	miR-30a-3p		[120]
	miR-34a		[110]
	miR-145		[120]
	miR-146a		[106]
	miR-132		[69]
	miR-152		[120]
	miR-155	TNF-α	[69]
	miR-194		[106]
	miR-203		[106]
	miR-302b	Bcl2, Cyclin D2	[110]
	miR-369		[106]
	miR-497	Bcl2	[110]
**Heart**	miR-138		[149]
	miR-485-5p		[149]
	miR-506		[149]
	miR-512-5p		[149]
	miR-548-3p		[149]
	miR-4262		[149]

Ethanol exposure alters the expression of microRNAs to fine-tune protein levels that are involved in different signaling pathways. We presented evidence for altered microRNA expression in all the organs reported suggesting that microRNA acts as an important mediator of alcohol-induced multi-organ damage. Ethanol-induced upregulated microRNA in vital organs and circulation were listed in Table 1 and downregulated microRNAs were listed in Table 2. Some of the microRNAs were similarly altered in two different organs and functions similarly. For example, miR-132 and miR-155 were elevated in liver, intestine and brain for a similar function to induce pro-inflammatory cytokines. Interestingly, microRNAs like miR-34a were elevated and exert dual role with ethanol exposure in two different organs. Increased miR-34a was shown to target caspase-2 and SIRT1 and acts as an anti-apoptotic signal in the liver with ethanol exposure. Whereas in the brain, increased miR-34a has a pro-apoptotic function. These observations suggest that more studies are needed to elucidate the mechanism behind this discrepancy. In addition, bioinformatics analysis of microRNAs suggests large number of target genes and it is important that these targets have been validated experimentally before drawing any conclusion. For example, ectopic overexpression of biotinylated miR-34a has been shown to target 982 genes [59]. However, how many of these miR-34a targets can be regulated by the endogenous levels of miR-34a remain unanswered [59]. Additional, microRNA target validation using RT-qPCR is necessary to confirm bioinformatics analysis or microarray results. In conclusion, we have presented evidence for the role of microRNAs in ethanol-induced multi-organ damage to pancreas, bone marrow, liver, intestine, brain, heart and muscle. Further, we have also presented evidence for ethanol as an epigenetic modulator during paternal and maternal ethanol consumption.

**Table 2:** Downregulated microRNAs with ethanol consumption. Several microRNAs were found to be decreased in pancreas, liver, brain and heart with ethanol exposure. Downregulated microRNAs were listed by affected organs, and their known target proteins were included with references.

Affected Organ	Downregulated microRNA	Targets	References
**Pancreas**	miR-148a		[18]
	miR-802		[18]
**Liver**	Let-7a		[24]
	Let-7b		[24]
	Let-7g		[24]
	miR-27b		[23]
	miR-122		[24]
	miR-127		[24]
	miR-27b		[23]
	miR-181a,b		[24]
	miR-182		[23]
	miR-183		[23]
	miR-192		[24]
	miR-199a-3p	Endothelin-1, HIF1-α	[23,25,32]
	miR-200a		[23]
	miR-214		[23]
	miR-322		[23]
	miR-871		[24]
**Brain**	miR-9		[94,123,124,125,126]
	miR-21		[79,80,81]
	miR-26a	BDNF, SIRT1	[97]
	miR-29a		[91]
	miR-29b		[91]
	miR-133		[91]
	miR-153		[79,80,81]
	miR-335		[126]
	miR-495	BDNF, SIRT1	[97]
**Heart**	miR-30a	Beclin 1	[148]

## Abbreviations

ALD	alcoholic liver disease;
ALT	alanine amino transferase;
BCL2	B-cell lymphoma 2;
MCL1	myeloid cell lymphoma 1;
BDNR	brain-derived neurotropic factor;
CB_1_R	cannabinoid receptor 1;
CCL2	chemokine (c-c motif) ligand 2;
CYP2E1	cytochrome p450-2E1 oxidase;
DR5	death receptor 5;
DGCR8	DiGeorge syndrome critical region 8;
RBC	red blood cells;
ROS	reactive oxygen species;
FAEE	fatty acid ethyl ester;
FASLG	FAS ligand G;
FoxM1	forkhead box protein M1;
LCFA	long chain fatty acids;
IL	interleukin;
lncRNA	long non-coding RNA;
LPS	lipopolysaccharide;
***MALAT1***	metastasis associated lung adenocarcinoma transcript 1;
PGC-1α	peroxisome proliferator-activated receptor 1 alpha;
RAR	retinoic acid receptor;
STAT3	signal transducer and activator of transcription 3;
SCFA	short chain fatty acids;
SOCS1	suppressor of cytokine signaling1;
SIRT	Sirtuins;
SREBP	sterol regulatory element-binding protein;
TLR4	toll-like receptor 4;
TGF-β	transforming growth factor-β;
TNF-α	tumor necrosis factor-α;
TBX5	T-box transcription factor 5;
UTR	un-translated region;
VLDL	very low density lipoproteins;
ZO-1	zona occludens.
